# The clinical role of the TME in solid cancer

**DOI:** 10.1038/s41416-018-0327-z

**Published:** 2018-11-09

**Authors:** Nicolas A. Giraldo, Rafael Sanchez-Salas, J. David Peske, Yann Vano, Etienne Becht, Florent Petitprez, Pierre Validire, Alexandre Ingels, Xavier Cathelineau, Wolf Herman Fridman, Catherine Sautès-Fridman

**Affiliations:** 1 Pathology Department, The Johns Hopkins University School Of Medicine, Baltimore, MD USA; 2 Urology Department, Institut Mutualiste Montsouris, F-75014 Paris, France; 3 Oncology Department, Hopital Européen Georges Pompidou, Paris, France; 4 INSERM, UMR_S 1138, Centre de Recherche des Cordeliers, Team “Cancer, immune control and escape”, F-75006 Paris, France; 5 University Paris Descartes Paris 5, Sorbonne Paris Cite, UMR_S 1138, Centre de Recherche des Cordeliers, F-75006 Paris, France; 6 Sorbonne University, UMR_S 1138, Centre de Recherche des Cordeliers, F-75006 Paris, France; 7 Singapore Immunology Network (SIgN), Agency for Science, Technology and Research (A*STAR), Singapore, Singapore; 8 Programme Cartes d’Identité des Tumeurs, Ligue Nationale contre le Cancer, F-75013 Paris, France; 9 Pathology Department, Institut Mutualiste Montsouris, F-75014 Paris, France; 10 University Paris Descartes Paris 5, Sorbonne Paris Cite, Paris, France

**Keywords:** Cancer microenvironment, Prognostic markers, Immunosurveillance, Cancer immunotherapy

## Abstract

The highly complex and heterogenous ecosystem of a tumour not only contains malignant cells, but also interacting cells from the host such as endothelial cells, stromal fibroblasts, and a variety of immune cells that control tumour growth and invasion. It is well established that anti-tumour immunity is a critical hurdle that must be overcome for tumours to initiate, grow and spread and that anti-tumour immunity can be modulated using current immunotherapies to achieve meaningful anti-tumour clinical responses. Pioneering studies in melanoma, ovarian and colorectal cancer have demonstrated that certain features of the tumour immune microenvironment (TME)—in particular, the degree of tumour infiltration by cytotoxic T cells—can predict a patient’s clinical outcome. More recently, studies in renal cell cancer have highlighted the importance of assessing the phenotype of the infiltrating T cells to predict early relapse. Furthermore, intricate interactions with non-immune cellular players such as endothelial cells and fibroblasts modulate the clinical impact of immune cells in the TME. Here, we review the critical components of the TME in solid tumours and how they shape the immune cell contexture, and we summarise numerous studies evaluating its clinical significance from a prognostic and theranostic perspective.

## Introduction

The tumour microenvironment (TME) is a highly complex ecosystem. Tumour cells co-exist with immune cells (including macrophages, polymorphonuclear cells, mast cells, natural killer cells, dendritic cells (DCs), and T and B lymphocytes) and non-immune cells (such as endothelial cells and stromal cells) and establish subtle interactions with them that determine the tumour’s natural history. In particular, the immune cell component of a tumour is fundamental in determining the tumour’s fate, and its invasive and metastatic ability. A large variety of immune cells can infiltrate tumours, and their composition and organisation within the TME are tightly associated with the clinical outcome of cancer patients. Current efforts to include immune parameters among the classical oncology prognostic classification tools have shown promising results.

The advancements in our understanding of the TME have also led, in recent years, to the development of efficacious therapies to treat advanced cancer. The treatment of thousands of cancer patients with monoclonal antibodies targeting inhibitory receptors expressed by immune cells (immune checkpoint blockade) has yielded remarkable response rates in several types of solid and haematologic malignancies.^1^ In this context, the analysis of the TME has become fundamental to predict response to treatment.

The constantly evolving knowledge about the complexity of the cancer niche and the dynamic interactions between all its components has changed the way we think about tumours. In this integrated model, the TME is shaped in situ by tumour and non-tumour cellular components, and by other elements such as the surrounding microbiota. In this article, we discuss evidence that supports the immune microenvironment as an essential player in this integrated cancer model, and we summarise numerous studies, evaluating its clinical significance from a prognostic and theranostic perspective in solid tumours.

## The TME

The neoplastic immune microenvironment is extremely complex, as virtually all immune cell types, including macrophages, polymorphonuclear cells, mast cells, NK cells, DCs, and T and B lymphocytes, can infiltrate cancer tissues.^2^ The role of these immune cell types in tumour evolution and growth is diverse and is tightly linked to their inherent functions and to the molecules they express (e.g., cytokines^3^ or inhibitory ligands).

The prototypical anti-tumour immune cell is the CD8^+^ T lymphocyte, which can recognise tumour cells in an antigen-specific manner and secrete cytotoxic molecules to kill them directly. Before exerting their cytotoxic functions, however, CD8^+^ T cells must be primed and educated by professional antigen presenting cells (APCs), i.e., DCs. Although these interactions between naive T cells and mature DCs cells have traditionally been thought to take place in secondary lymphoid organs (i.e., lymph nodes), it is now clear that they can also occur within, or adjacent to, the tumoural tissue, in organised tertiary lymphoid structures.^4^ These specialised structures provide an area within the TME that is protected from the immunomodulatory effects of the tumour or stromal cells, and is enriched with T cell activation cytokines. Recent studies suggest that the anti-tumour immune response can be orchestrated within these structures.^5,6^ Cytotoxic NK cells can also exert anti-tumour killing independently of any previous interaction with APCs,^7^ notably in the case of a loss of class I major histocompatibility complex (MHC) molecules on the surface of tumour cells. CD4^+^ Th1-oriented T cells are also important in promoting an anti-tumour immune response through the production of cytokines essential for T cell proliferation, as well as for macrophage recruitment and activation.^8^

By contrast, other immune cells, such as tumour-promoting M2 macrophages and immature granulocytic and monocytic cells (myeloid-derived suppressor cells (MDSCs)) can favour tumour progression through the induction of stromal cell proliferation, vascularisation, extracellular matrix deposition (ECM), and cell migration.^9–11^ These and other immune cells can also promote tumour progression by inhibiting the in situ immune response. The prototypical immunosuppressor cells are regulatory CD4^+^ T lymphocytes (Treg), which directly secrete or facilitate the formation of immunosuppressive molecules (e.g., IL-10, adenosine), and modulate the APC function (e.g., via CTLA-4–CD80/86 interactions.)^12^

Some immune cells often demonstrate plasticity in the TME, showing both tumour-promoting and tumour-inhibiting potential. For example, whereas some macrophages (M1) mainly produce pro-inflammatory cytokines that potentiate the anti-tumour immune response, others (M2) can promote fibroblast proliferation, ECM deposition and immunosuppression.^13^

Importantly, the total composition of immune cells in the TME is not a binary ‘anti-tumour’ or ‘pro-tumour’ environment, but rather a mixture of these cell types. The overall phenotype is determined by the interactions of the immune cells with each other and with non-immune cells. The tumour and stromal cells play an essential role in determining the composition of the immune cell infiltrate, as well as the rate of recruitment, and their in situ phenotype and function.

## Shaping the TME

### The tumour mutational landscape

**Fig. 1:**
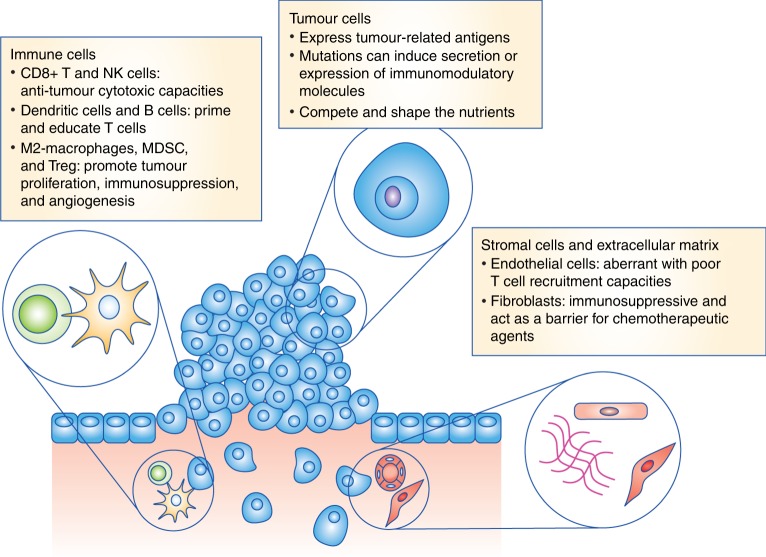
The tumour microenvironment and immune contexture in cancer. Schematic depicting the three main components in the tumour microenvironment. The mechanisms shaping the immune cell contexture are highlighted. MDSCs, myeloid-derived suppressor cells; Treg, regulatory CD4^+^ T cells

Genetic mutations, ranging from single base substitutions to chromosome translocations, are the cornerstone of the development of neoplastic lesions.^14^ Individual DNA mutations that accumulate over time often lead to the stepwise transformation of normal cells into dysplastic and eventually neoplastic tumour cells. These mutations can be induced by a broad array of cellular stressors, such as carcinogens in cigarette smoke, ionising radiation including UV light from the sun, or reactive oxygen species generated by myeloid cells during chronic inflammation.^15^ The number and characteristics of these pathogenic tumour mutations shape the composition of the immune microenvironment through several different mechanisms (Fig. 1).

First, a portion of mutated peptide epitopes resulting from either driver or passenger mutations can be presented on tumour cell class I MHC molecules and be recognised by CD8^+^ T cells, which, together with T-cell-attracting chemokines such as CXCL9 and CXCL10, promotes a brisk infiltration by cytotoxic T lymphocytes. The mutations that create tumour-associated neoantigens have become more appreciated with recent advances in sequencing and computational techniques, leading to the concept that a tumour mass is often composed of different subclones of cells with different immunogenic potentials.^16,17^ The prototypical example of immune cell response induced by tumour cell mutations is microsatellite instable (MSI) colorectal cancer (CRC). This subtype represents ~ 15% of CRC cases and is characterised by defective DNA mismatch repair machinery, resulting in an increased rate of mutagenesis as compared with microsatellite stable tumours (MSS).^18^ Furthermore, MSI CRC tumours display increased infiltration with CD8^+^ T cells, B cells and macrophages, as well as an increased expression of Th1-related genes.^19,20^

Second, some driver or passenger mutations can induce molecular pathways that shape tumour infiltration by immune cells independently of their neoantigenic potential. For example, the mutation-driven activation of the Wnt-β-catenin pathway in melanoma,^21^ colorectal cancer,^22,23^ and hepatocellular carcinoma^24^ limit the accumulation of cytotoxic T cells and DCs. In addition, models of lung adenocarcinoma suggest that mutations in the Myc and Ras pathways cooperate to establish an immunosuppressive microenvironment by driving expression of the chemokine CCL9—which recruits immunosuppressive and angiogenic macrophages – and interleukin (IL)-23, and prevents the accumulation of cytotoxic NK cells and T cells in the tumour.^25^ Similarly, KRAS mutations in pancreatic ductal cells drive the expression of granulocyte-macrophage colony-stimulating factor, which leads to the recruitment of large numbers of neutrophils and prevents an effective anti-tumour T cell response.^26^ It has been shown that other primary tumours, including those in the breast, lung and gastrointestinal cancer, also activate granulopoiesis in the bone marrow and actively stimulate the release and recruitment of mature immunosuppressive neutrophils and monocytes from the circulation.^27–29^

Third, mutations in tumour cells can alter immune cell functions once they are recruited within the tumour mass. For example, Coelho et al. found that oncogenic RAS can drive tumour cell-intrinsic upregulation of the programmed cell death ligand 1 (PD-L1).^30^ By binding to programmed cell death protein 1 (PD-1) expressed on activated T cells, PD-L1 suppresses the effector activity of T cells that might otherwise confer cytotoxicity.

**Fig. 2:**
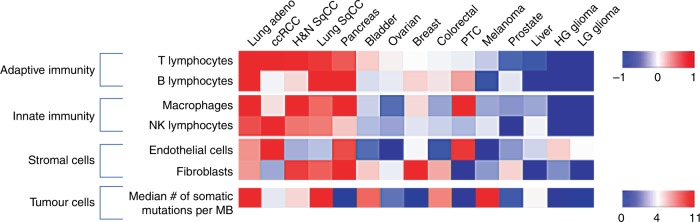
The tumour microenvironment and mutational landscape across tumour types. Heatmap representing the average number of somatic mutations (ranging from 0.7 per megabase in thyroid cancer, to 11 per megabase in melanoma)^155^ and relative abundance of infiltrating immune and stromal cells across 15 different human tumours as determined by Microenvironment Cell Populations-counter (MCP-counter).^156^ The cancer types are organised from left to right according to the abundance of T cell infiltrate. SqCC, squamous cell carcinoma; Adeno, adenocarcinoma; H&N, head and neck; ccRCC, clear cell renal cell carcinoma; LG, low-grade; HG, high-grade; PTC, papillary thyroid cancer

These three mechanisms shape the composition of immune cells within the tumour significantly. In some tumour types, such as lung adenocarcinoma, the extent of T cell infiltration is tightly correlated with neoantigen frequency.^31^ However, no such correlation has been observed in melanoma patients.^32^ Other tumours, such as pancreatic adenocarcinoma and renal cell carcinoma (RCC), have a lower frequency of neoantigens but might still exhibit a high degree of infiltration by T cells (Fig. 2). Thus, although the mutational load is tightly correlated with the degree of tumour inflammation, the nature of the driver mutations and additional neoantigenic mutations that accumulate critically influence the nature and function of the immune cell composition.

### The non-immune components

Tumour cells have high mitotic and metabolic rates and therefore need to maintain sufficient oxygen and nutrient levels to support their growth. A vascular network is fundamental for a tumour’s development. The hypoxic tumour environment promotes the production of proangiogenic factors (e.g., vascular endothelial growth factor, transforming growth factor-β, fibroblast growth factor and platelet-derived growth factor.),^33,34^ which can prompt rapid angiogenesis but often results in the formation of aberrant vasculature.^35^ Several studies have shown that tumour-associated endothelial cells often express low levels of leucocyte adhesion molecules (e.g., ICAM1 and VCAM1) and T cell-recruiting chemokines, thereby impeding the recruitment of anti-tumour immune cells.^36–39^ Furthermore, the tumour blood vessels are tortuous, leaky and with reduced pericyte coverage, which might present mechanical barriers to infiltration by T cells.^40^

Conversely, in situations where the tumour vasculature does contain appropriate ligands, an active T cell infiltrate can be present. Of particular interest is the development of tertiary lymphoid structures (TLSs), which are highly specialised immune aggregates.^4,41,42^ These immune aggregates contain not only a high density of immune cells facilitating their interactions but also a highly efficient and specialised vasculature–so-called ‘high endothelial venules,’ which are otherwise only found in lymph nodes. These specialised vessels are capable of actively recruiting naïve T cells and B cells from the circulation. Mature DCs that migrate to the site activate and educate the newly recruited T cells, which proliferate and differentiate into T effector memory cells. The B cells also proliferate and differentiate into plasma cells. Thus, although many characteristics of the tumour vasculature hinder the development of an immune cell composition that is predominantly ‘anti-tumour,’ certain cases, such as the formation of TLSs and infiltration by T^6^ and B^43^ cells, can support an adaptive anti-tumour immune response.

Of the other non-endothelial tumour-associated stromal cells, fibroblasts have been most convincingly shown to shape the process of immune cell recruitment and differentiation. Various studies suggest that under certain stimuli, cancer-associated fibroblasts (CAF) can acquire a pro-inflammatory signature characterised by the expression of immunomodulatory molecules (e.g., TGF-β^44^ or PD-L1/L2^45,46^), as well as chemokines that promote recruitment of immunosuppressive myeloid cells (e.g., CXCL12, CCL2, CCL3, CCL4 and CCL5).^9,47,48^ In addition, CAF often form part of an intricate and thick arrangement of cells and stromal matrix surrounding tumour nests (also known as a desmoplastic reaction), which represents a physical barrier for cytotoxic immune cell infiltration.^49^

Finally, by shaping the balance of nutrients and oxygen within the TME, the tumour cell can also actively modulate the phenotype and function of the infiltrating immune cells.^50,51^ Several studies have provided evidence that the function of the immune cells, in particular T cells, is tightly linked to their metabolic state and the abundance of certain nutrients. For instance, the depletion of glucose or arginine impair T cell proliferation and cytotoxic functions.^52,53^ The consumption of nutrients and the expression of certain enzymes by the tumour cells can thus deeply impact the TME. Chang et al.^54^ showed that the consumption of glucose by tumour cells metabolically restricts the infiltrating T cells, leading to dampened IFN-γ production. Also, the overexpression of indoleamine 2,3-dioxygenase (rate-limiting enzyme of tryptophan catabolism) by the tumour cells has been associated with the inhibition of T cell functions.^55^

### The unexpected players: the gut microbiota

Recent evidence has highlighted that the microbiota can have a significant effect on the rate of tumour growth and spread, probably by shaping the systemic and in situ immune response. Within the human body, several organs—including the skin, gut and other mucosas–are colonised by several trillions of microbes, which constantly interact with the host. This interaction has relevant systemic effects, including shaping the functional diversity and the repertoire of B and T cells.^56^ Although certain commensal bacteria seem necessary to maintain a tonic baseline immune response,^57,58^ others can induce a systemic immunoregulatory effect.^58^ For example, alterations in the gut microbiota are associated with HIV disease progression.^59^ In contrast, several studies suggest that certain bacteria can induce a local and systemic expansion of Treg that are essential to prevent tissue inflammation.^60–65^

Not surprisingly, alterations in the gut microbiota also have effects on cancer development and spreading. For instance, it has been shown that hepatocellular carcinoma is promoted by the intestinal microbiota via activation of TLR4.^66^ The area with the most interesting evidence is currently cancer therapeutics. In mice, two studies showed that chemo-^67^ and radiotherapy^68^ promoted gastrointestinal bacterial translocation into the systemic circulation, which probably boosts the immune response and promotes post-therapy tumour rejection. Similarly, Lida et al.^69^ showed that the CD8^+^ T cell-driven tumour rejection induced by intratumoural CpG-oligodeoxynucleotides is inhibited by antibiotics.

In the area of checkpoint blockade, recent studies have shown promising evidence that the response to these agents is modulated by the gut microbiota. Vetizou et al.^70^ showed that the anti-tumour effects of CTLA-4 blockade depends on distinct Bacteroides species. In this study, tumours in antibiotic-treated or germ-free mice did not respond to CTLA-4 blockade; this defect was overcome by gavage with *Bacteroides fragilis*, by immunisation with *B. fragilis* polysaccharides, or by adoptive transfer of *B. fragilis*-specific T cells. Similarly, three recent articles described similar findings with anti-PD-1/PD-L1 therapy. Routy et al.^71^ showed that fecal microbiota transplantation (FMT) from cancer patients who responded to anti-PD-1 into germ-free or antibiotic-treated mice established the anti-tumour effects of PD-1 blockade, whereas FMT from non-responding patients failed to do so. Similar results were found by Matson et al.^72^ in melanoma patients treated with anti-PD-L1. Finally, Gopalakrishnan et al.^73^ found that responding patients with a ‘favourable gut microbiome’ showed an enhanced systemic and anti-tumour immunity after anti-PD-1 treatment.

## The immune cell contexture as a prognostic tool in modern clinical practice

The analysis of the immune microenvironment in retrospective cohort studies across different tumours has established a clear correlation between the density of infiltrating immune cells and the patient’s clinical outcome. More than 280 articles assessing the correlation between the presence of distinct immune cell populations and patient prognosis have been published to date (reviewed in detail in ref.^74–76^). Overall, clear-cut evidence has established that the presence of the main cellular players orchestrating the cytotoxic anti-tumour immune response (e.g., cytotoxic CD8^+^ T cells, Th1-oriented CD4^+^ T cells, mature activated DCs and TLSs) is associated with a good clinical outcome in the vast majority of tumour types. In contrast, high densities of macrophages—specifically M2-oriented—and Tregs are associated with poor prognosis. The major efforts to include quantification of these immune populations in the standard clinical practice have been conducted in melanoma and colorectal cancer, as outlined below.

There are a few rare examples of tumours that do not follow the association between high infiltration with CD8^+^ T cells and a positive prognosis. RCC has been the best studied of these examples to date, and a relevant number of studies suggest that increased densities of CD8^+^ cells are associated with patients’ shorter survival. Recent evidence highlights that this unexpected correlation is probably related to a dysfunctional immune cell response in this tumour type, and to the expression of inhibitory receptors by tumour-infiltrating T cells.^77,78^ In node-positive prostate cancer, a stronger infiltration by CD8^+^ T cells has also been associated with an enhanced risk of metastasis.^79^ This paradoxical association has also been described in some haematologic malignancies (e.g., diffuse large B-cell lymphoma^80^ and Hodgkin lymphoma^81^).

### Melanoma

Melanoma was one of the first tumour types in which a high density of tumour-infiltrating lymphocytes (TILs) was found to correlate with favourable patient prognosis, including a lower incidence of lymph node metastasis and longer disease-free survival (DFS).^82,83^ Pioneering studies by Azimi et al.^84^ and Thomas et al.^85^ generated data from > 4000 patients with melanoma, semi-quantitatively grading their lesions according to the degree of lymphocyte infiltration on Hematoxylin and eosin-stained slides. This grading scheme (grade 0, TILs absent; grade 1, either a mild or moderate focal or a mild multifocal lymphocyte infiltrate; grade 2, a marked focal, either a moderate or marked multifocal, or a mild diffuse lymphocyte infiltrate; and grade 3, a moderate or marked diffuse lymphocyte infiltrate) was an independent predictor of DFS, such that a lower grading correlated with a lower DFS. Notably, patients with tumours assessed to be TIL grade 3 showed 100% survival after 5 years. These and other studies^86,87^ have helped the TIL grading system win recognition among clinicians as a feasible and inexpensive prognostic factor in patients with melanoma, and it is now routinely reported by pathologists following the recommendations of the College of American Pathology (cancer protocol templates for melanoma^88^). This approach supplements other pathologic parameters that predict patient prognosis, including tumour, node and metastasis (TNM) staging, the presence of vascular invasion or tumour regression and the mitotic rate.

### Colorectal cancer

Another big effort to validate the prognostic significance of the presence of TILs has been led in CRC, in which an immunohistochemistry (IHC)-based grading score system called Immunoscore has been optimised and developed.^89^ This process involves scanning a stained slide and analysing it using digital pathology IHC quantification software. Under the Immunoscore grading system, CD3^+^ and CD8^+^ cells in the invasive margin and the internal core of the tumour are quantified (as cells/mm^2^) and scored as low, intermediate, or high. These groups were defined using cell density cutoffs based on the mean distribution of CD3^+^ and CD8^+^ cells in 700 tumour lesions (0–25% low, 25–70% intermediate and 70–100% high). An international consortium has scored the tumours of > 3500 CRC patients according to this grading system and has determined that it has a prognostic significance superior to that of the classical TNM system.^90,91^ Interestingly, a recent study^20^ evaluating T cell infiltrate in CRC tumours found that although MSI CRC display on average a higher immune infiltrate as compared with MSS, a significant fraction still displayed a low Immunoscore. There were also several MSS tumours that exhibited a high Immunoscore. Based on this evidence, the authors suggested that the Immunoscore was superior to microsatellite instability in predicting patient disease-specific recurrence and survival.

Although the evidence associating the Immunoscore with clinical outcome is strong, there are several factors that challenge the implementation of this quantification system in clinical practice. To score a given tumour, an automated system for IHC staining is required, together with a high-resolution slide scanner and digital pathology software.

### RCC

Contrary to most tumours in which increased numbers of CD8^+^ T cells are associated with a favourable clinical outcome, the opposite association has been described for RCC. Our research group has studied this paradoxical association in detail. Our data suggested that the increased infiltration with CD8^+^ T cells in RCC can be accompanied by either a well orchestrated or an immunosuppressive TME, and this context determines the patient’s prognosis. Although two-thirds of the tumours with high densities of CD8^+^ TIL are associated with low infiltration by mature DCs, increased expression of inhibitory ligands (e.g., PD-L1 and PD-L2) and poor clinical outcome, the remaining third is highly enriched in TLSs, exhibit proliferating T cells and is associated with remarkably long PFS.^77,92^

**Fig. 3:**
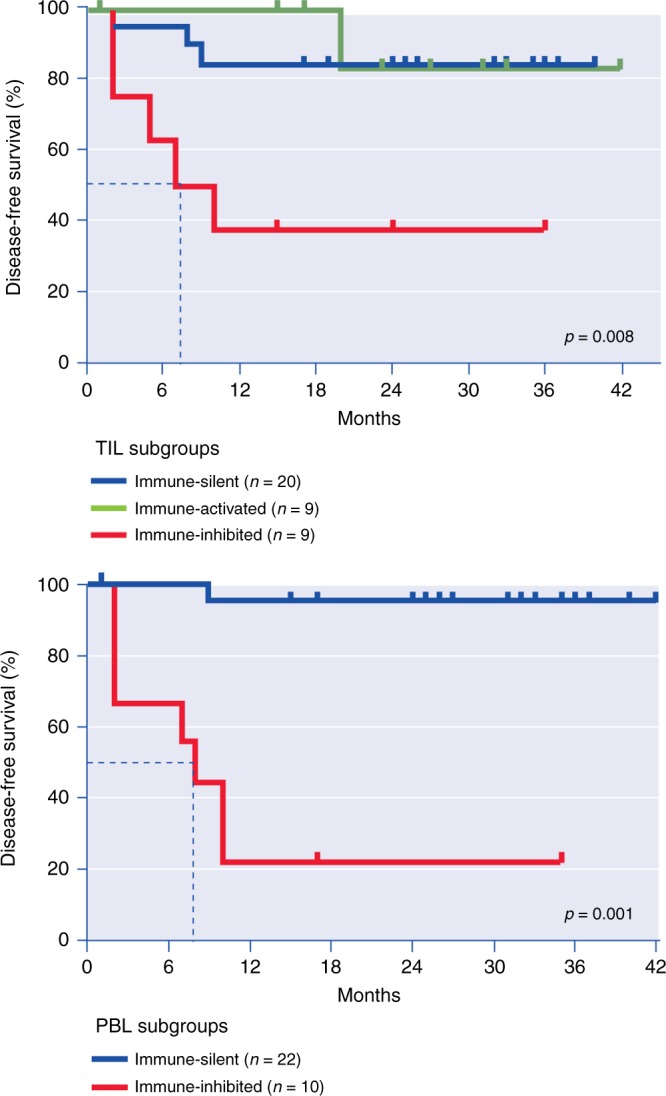
Intra-tumour and peripheral blood T cell immune profiles and prognosis in ccRCC. Disease-free survival according to tumour-infiltrating lymphocyte (TIL) and peripheral blood lymphocyte (PBL) subgroups. *P* values according to univariate Cox regression analysis are displayed. Updated April 2018 from reference^78^

Altogether these data supported the existence of three different immune profiles in RCC: one with activated fully functional T cells (immune-activated): one with abundant but inhibited T cells (immune-inhibited); and one with low infiltration by any immune cell type (immune-silent).^2,93,94^ To prove this concept, we used multiparametric flow cytometry to analyse the phenotype of tumour-infiltrating T cells in 38 patients with clear cell renal cell carcinoma (ccRCC) (T stage 1–3), and followed the patients prospectively for one year (median follow-up 11 months ± 6.)^78^ We investigated the co-expression of 14 activation molecules and inhibitory receptors and subclassified the tumours by unsupervised methods using phenotype data. We corroborated the existence of three immune profiles in RCC and determined that patients with immune-inhibited tumours had an extremely high risk of recurrence in the first year after surgery (70%). These tumours were characterised by the presence of abundant regulatory CD4^+^ T cells and CD8^+^ T cells co-expressing several inhibitory receptors (e.g., PD-1, Tim-3 and Lag-3). We have recently updated the PFS data for this prospective cohort (median follow-up of 26 months ± 6, Fig. 3) and confirmed the significant differences in the PFS between patients with these three groups of tumours. The median survival of patients with immune-inhibited tumours was only 8 months, but it has not yet been ascertained for individuals with immune-silent or immune-activated tumours (Fig. 3).

We also analysed phenotypic T cell markers in the peripheral blood lymphocytes of this group of ccRCC patients and, through unsupervised methods, were able to define two main groups of patients: peripheral blood lymphocyte (PBL)-immune-silent, with almost absent expression of activation markers (e.g., CD69 and inducible T cell co-stimulator] or inhibitory receptors (e.g. PD-1, Tim-3 and CTLA-4); and PBL-immune-inhibited, with prominent expression of activation markers and inhibitory receptors. The updated follow-up of these patients showed a sharp difference in their PFS (Fig. 3). Although the disease has progressed in almost 80% of the patients with PBL-immune-inhibited after 24 months, this number only reaches 10% in the PBL-immune-silent group. This is a relevant finding given the feasibility of analysing the expression of phenotypic markers in PBL from cancer patients. These promising results are currently being investigated in prospective clinical trials to evaluate its significance as prognostic and theranostic tools.

### Other tumours

Although not always exhaustively studied in the clinical setting, other solid malignancies deserve particular attention given the abundant evidence associating the TME with clinical outcome.

In breast cancer, the analysis of thousands of samples has found a strong association between high infiltration with CD8^+^ T cells or a Th1-gene signature and longer PFS and OS.^95–100^ Also, it has been suggested that this association is particularly strong in oestrogen receptor (ER) negative, HER-2 negative, as well as ER, progesterone receptor, HER-2 triple-negative breast cancers.^99^ In contrast, the infiltration with macrophages is associated with poor prognosis.^101–103^

In non-small cell lung cancer (NSCLC) the infiltration with CD8^+^ cells has been associated with good clinical outcome in several studies that have included several thousands of patients.^6,104–109^ Interestingly, Goc et al.^6^ found that lung tumours with high infiltration with CD8^+^ cells but low densities of mature DCs were associated with poor prognosis, as compared with tumours with high numbers of both populations. Also, some studies have associated the densities of macrophages and B cells with extended survival in patients with NSCLC.^43,105,110–113^

## The immune cell contexture as a theranostic tool in the checkpoint blockade era

The expression of inhibitory receptors (e.g., CTLA-4, PD-1, Lag-3) by tumour-infiltrating lymphocytes cells has gained significant attention in recent years in the oncology field. Many of these molecules are expressed on T and B cells upon activation, and their physiologic role is to inhibit the immune function once they bind to their respective ligand.^114^ Several clinical trials using monoclonal antibodies to block these receptor-ligand interactions have shown remarkable response rates in solid cancer and haematologic malignancies in recent years. The sensitivity to these therapies seems to depend on many factors, including some intrinsic features of the TME.^115^

The clinical impact of PD-1-PD-L1/L2 blockade in cancer has been extensively studied. To date, data for thousands of patients have been reported, with durable objective response rates (ORR) ranging 32–42% in melanoma, 12–26% in NSCLC, 14–31% in urothelial cancer and 14–21% in RCC. Biomarkers to predict clinical outcome have also been studied in many of these trials. The increased expression of PD-L1 by tumour or infiltrating immune cells, high mutational loads and increased densities of TIL, are the most promising biomarkers that best correlate with response to therapy.

### PD-L1 expression

The first two clinical trial using anti-PD-1 agents (nivolumab and atezolizumab) in patients with solid tumours (melanoma, NSCLC, RCC, head and neck, prostate, breast and colorectal cancer) suggested that the expression of PD-L1 in pre-treatment specimens (defined as > 5% tumour cell expression) was associated with response to treatment.^116,117^ Subsequently, the majority of clinical trials assessing response to PD-1–PD-L1 blockade have evaluated the protein expression of PD-L1 by the tumour or infiltrating immune cells and established its association with clinical outcome.^1^ Although the general pattern is consistent, the absolute response rates and strength of the association between the expression of PD-L1 and response to PD-1/PD-L1 blockade varies across tumours. In melanoma (nivolumab^118–122^) numerous clinical trials have reported significantly higher ORR in patients with PD-L1^+^ tumours (~ 53%) vs. PD-L1^-^ (~ 34%). In NSCLC (nivolumab,^123–127^ pembrolizumab,^128,129^ atezolizumab,^117,130^ and avelumab^131^) the ORR is also higher in patients with PD-L1^+^ tumours (30%) vs. PD-L1^-^ (19%). Similar percentages have been reported in urothelial cancer (nivolumab,^132^ atezolizumab,^133–135^ durvalumab,^136,137^ or avelumab^138^), RCC (nivolumab^139^ and atezolizumab^140^), head and neck squamous cell carcinoma (pembrolizumab^141^) and gastric cancer (pembrolizumab^142^). Given this association, the FDA has approved the use of some of these therapeutic agents only in patients exhibiting a certain degree of PD-L1 expression on their tumour.^89^

### Mutational loads

Early evidence suggesting that the extent of DNA damage could also be correlated with response to checkpoint blockade came from the fact that cancers that demonstrated the highest response rates to PD-1/PD-L1 blockade therapies (e.g., melanoma, NSCLC, bladder and stomach cancer) displayed the highest mutational loads among all tumour types.^143,144^ Furthermore, lesions associated with mutagenic aetiologies (e.g., smoking in NSCLC) or displaying defects in their DNA-repairing machinery (e.g., MSI) exhibited higher ORR to anti-PD-1 treatments than tumours from other aetiologies.^145,146^ In addition, mismatch repair deficient tumours are particularly sensitive to anti-PD-1 therapy.^147^

The quantification of tumour mutational loads has been included in some clinical trials evaluating the activity of PD-1/PD-L1 blockade and has revealed its potential association with therapeutic response. In patients with NSCLC and treated with pembrolizumab, some tumoural non-synonymous mutations above the cohort’s median have been associated with higher ORR and longest PFS.^145,148^ Interestingly, these studies have also suggested that patients with PD-L1^+^ tumours and high mutational burdens exhibit the highest sensitivity to anti-PD-1 therapy (ORR 91%). These findings were supported by a clinical trial in urothelial cancer where the activity of atezolizumab was tested; responding patients exhibited two-fold higher mutational loads than non-responding patients.^134^ Finally, a study in melanoma-bearing patients receiving pembrolizumab (*n* = 38) found that high mutational burdens, although not associated with clinical response, correlated with improved survival.^149^

### Density of TIL

Some clinical trials have also evaluated the correlation between TIL density and clinical response to anti-PD-1/PD-L1 therapies. Chen et al.^150^ reported higher densities of CD3^+^, CD8^+^ and CD45RO^+^ memory TIL in the pre-treatment biopsies from patients with melanoma who responded to nivolumab than non-responders. A similar association between CD8^+^ TIL numbers and response to treatment has been reported in patients with melanoma and colorectal cancer treated with pembrolizumab^151,152^ or atezolizumab.^140^ By contrast, two independent studies have reported that CD8^+^ TIL densities are not associated with response to anti-PD-1 in metastatic RCC.^140,153^

## Conclusions

During the past two decades our understanding of the complexity of the TME has increased considerably. In-depth characterisation of each of the cellular components in cancer has shed light on the convoluted network of interactions between the numerous components within a tumour mass. Furthermore, the clinical follow-up of large cohorts of cancer patients has shown how these interactions have largely determined the clinical evolution of cancer. The addition of immune-based prognostic biomarkers to the current clinical practice of oncology and pathology is imminent, as it rapidly gains recognition within the medical community.^89^ The next generation of tumour histology-based predictive biomarkers will probably transcend single-stain IHCs, as the whole research field is rapidly moving toward multiparametric and highly complex techniques (i.e., multispectral immunofluorescence, spatial transcriptomics and mass-spectrometry-based tissue imaging.)^154^ The challenge facing the tumour immunology community is how to translate these new highly complex findings into relevant, simple and consistent biomarkers to use in the clinical setting.
